# Genetic segregation for male body coloration and female mate preference in the guppy

**DOI:** 10.1186/s13104-020-4909-5

**Published:** 2020-01-30

**Authors:** Aya Sato, Masakado Kawata

**Affiliations:** 1 Faculty of Education, Gunma University, 4-2 Aramaki, Maebashi, Gunma 371-8510 Japan; 2 Graduate School of Life Sciences, Tohoku University, Aoba-ku, Sendai, 980-8578 Japan

**Keywords:** Artificial selection, Female mate preference, Genetic variation, Guppy, Male coloration, Sexual selection

## Abstract

**Objective:**

The purpose of this study was to segregate the genetic lines responsible for the orange area of coloration in males and the response to orange coloration exhibited by females in the guppy (*Poecilia reticulata*) through artificial selection. This study is part of a project that uses QTL-seq to search for candidate genes involved in male orange coloration and female response to male coloration. We created two lines: high-selected lines of males having large areas of orange spots and of females with high response to male orange coloration; and low-selected lines of males having small areas of orange spots and of females with low response to male orange coloration.

**Results:**

The male orange area and the female response became significantly different between high- and low-selected lines after three generations of artificial selection. This indicates that the differences in the frequencies of alleles at loci affecting the orange area and the female response between the lines increased over the generations through selection.

## Introduction

The guppy, *Poecilia reticulata*, is a model fish for studies on sexual selection [1]. Female guppies generally prefer mating with males that possess larger and more colorful orange spots [2, 3]. However, substantial variations in both male coloration and female preference are maintained between and within populations [4–8]. A better understanding of the mechanism underlying this diversity would be gained from identifying the associated genes. Early genetic studies of male coloration variations have shown a strong patrilineal inheritance of large genetic components that determine male color patterning [9, 10]. The guppy possesses an XY sex-determination system, and extensive, traditional studies have shown at least 20 exclusively Y-linked color pattern alleles and at least 28 alleles that recombine between X and Y [11–14]. A few more recent studies using ornamental guppy strains have also shown genetic mapping of different color patterns to these sex chromosomes [15–17]. In addition, quantitative trait locus (QTL) studies for sets of variable male traits, including size and color pattern, have been performed using the linkage data of 790 single-nucleotide polymorphism (SNP) markers [18]. These results suggest that several loci located at different linkage groups affect the occurrence and size of orange spots. To date, however, this exact SNP marker has not yet been determined.

In the guppy, artificial selection experiments for increased and decreased male orange area have shown high and low selection lines with a strong divergence [19–21]. If male orange coloration and female preference for male orange coloration are genetically correlated, it is predicted that female preference for male orange areas occur due to artificial selection. The results of previous studies have supported this prediction [21, 22], although the results from another study has not [20]. While genetic studies on the variation of male coloration have accumulated, no specific sequence variant affecting female preference for male coloration have been determined, although variations in the expression of opsin genes is known to affect the female’s response to male coloration [23].

Recently, genome-wide association analysis has been used to identify the regions affecting the phenotypes [24–26]. Usually, a large number of individuals are required to detect significant SNPs. QTL-seq is another genome-wide method which uses phenotypically different breeding lines for identifying QTL loci [27]. We set up a project that uses QTL-seq to search for SNPs involved in both male orange coloration and female preference to orange coloration. In preparation, we artificially selected for male orange coloration and female preference to orange coloration, and created lines with opposite traits: males with large and small orange area and females with high and low preference to orange coloration. In this paper, we report on the results of the artificial selection.

## Main text

### Methods

#### Fish

Feral guppies were collected in March 2012 from 5 sites on Okinawa Island, Japan: Isa River, 26° 61′ N, 128° 00′ E; a water channel in Gabusoka, 26° 62′ N, 128° 00′ E; a spring pond in Inoha, 26° 66′ N, 127° 91′ E; Hiji River, 26° 72′ N, 128° 18′ E; and Okuma River, 26° 73′ N, 128° 17′ E. The caught guppies were transported to the University of Tohoku in Sendai, Japan, and kept in plastic tanks. After 1 month, once the fish had become accustomed to laboratory conditions, females were isolated in plastic tanks (2 L) and allowed to give birth. Thirty-one females produced broods (1 Isa, 1 Inoha, 2 Gabusoka, 11 Hiji, 16 Okuma). In addition, two females from a laboratory strain of feral guppy from Okinawa Island were isolated and allowed to give birth. In total, 85 male (P male) and 82 female (P female) offspring were collected from the 33 broods. These offspring were reared in plastic tanks (7 L) with males and females kept separately. After 6 months, male orange coloration and female mate preferences were measured.

All fish were maintained under constant conditions (25±1 °C, aerated and filtered water, 12:12 h light:dark cycle), and fed once daily with newly hatched brine shrimp (*Artemia salina*) nauplii and commercial flake food (Tetramin, Tetra Werke).

#### Male coloration and female preference

Male orange area was measured as the ratio of the total area of orange spots to the total area of the body and caudal fin following the procedure in Additional file 1. We conducted dichotomous choice tests using two video images of male, one of which was of a male with large/colorful orange spots (high-orange, HO) and the other was of a male with small/drab orange spots (low-orange, LO) to quantify female preference for male orange coloration. A detailed description of the procedure can be found in Additional file 1. The female preference to HO was calculated as the proportion of time that the female spent viewing the HO male image in relation to the total time that the female spent on both the HO and LO male images.

#### Artificial selection

Fifteen males with the largest orange area and 15 males with the smallest orange area were selected from the P males as the high- and low-selected lines, respectively. Fifteen females with the highest preference (from the female preference test) and 15 females with the lowest preference were selected from the P females for high- and low-selected lines, respectively.

A male and a female were selected from each line and paired such that males and females from the same broods did not mate. One female was placed into a plastic tank (2 L) with her paired male and they were maintained until the female produced a brood (F1). To ensure that all offspring were reared at the same density, four male and four female offspring were randomly selected after sex discrimination and reared separately each sex under the same conditions as the P individuals. Once these offspring had reached 6 months of age, the male coloration and the female preference were measured. Next, males and females were selected from the F1 generation and paired in the same way as for the P generation. The process and measurements were repeated until generation F3.

#### Statistical analysis

The male orange area and the female preference between selected lines were compared using *t*-test on independent samples in each generation. The *t*-test with assumed heteroscedasticity was used because variances of male orange area between selected lines were not homogeneous (Levene’s test; *F *> 10.78, *P* < 0.01).

Realized heritability (*h*^*2*^) was calculated separately for the low- and high-selected lines for male orange area and female preference as:

$$h^{2} = 2\,\left( {\frac{R}{S}} \right)$$

where *R* represents the cumulative response (the sum of means in generation *i* minus means in generation *i* − 1) and *S* represents the cumulative selection differential (the sum of selected individuals’ means in generation *i* minus all individuals’ means in generation *i*) [28]. The slope of the regression of *R* on *S* was estimated, with the multiplier of 2, and was used as selection for each trait for the separate sexes. Statistical analyzes were performed using SPSS version 25.0.

### Results

**Fig. 1:**
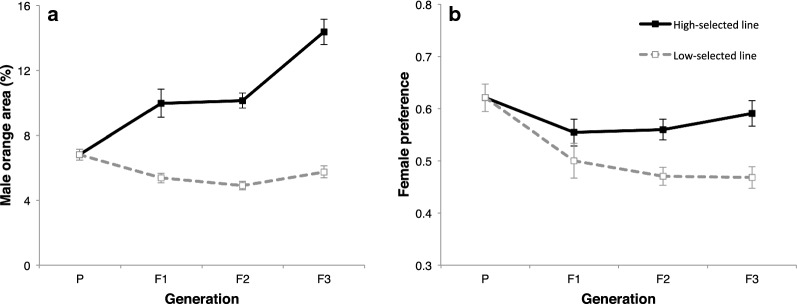
Responses of male orange area **a** and female preference **b** to artificial selection. Plots are mean ± SEM

In the high-selected lines, the orange area for 53, 54, 45 males and mate preference for 50, 55, 45 females were measured at F1, F2, F3, respectively (Additional file 2: Table S1). In the low-selected lines, the orange area for 47, 51, 45 males and mate preference for 48, 53, 47 females were measured at F1, F2, F3, respectively (Additional file 2: Table S1). After the three generations of artificial selection, the mean male orange area of the high-selected line increased 110.8% and that of the low-selected line decreased 15.6%, compared to those of P males (Fig. 1). The male orange area differed significantly between high- and low-selected lines in all generations (F1: *T*_64.710_ = 5.045, *P* < 0.001; F2: *T*_82.897_ = 9.970, *P* < 0.001; F3: *T*_64.072_ = 10.025, *P* < 0.001). The realized heritability of male orange area estimated from the high-selected line was significantly larger than zero, whereas that from the low-selected line was not (Table 1, *h*^2^ for each generation is shown in Additional file 2: Table S2).

**Table 1:** Realized heritability of male orange area and female preference. *P* indicates probability from one-sample t-test for difference to zero

	*h*^2^ ± SEM	*P*
Male orange area		
High line	1.472 ± 0.243	0.026
Low line	0.315 ± 0.111	0.105
Female preference		
High line	− 0.122 ± 0.120	0.416
Low line	0.595 ± 0.148	0.057

After the three generations of artificial selection, the mean female response to HO in high- and low-selected lines were 4.8% and 24.6% lower, respectively, than that of P females (Fig. 1). The female response to HO in high- and low-selected lines did not differ in F1 (*T*_83.483_ = 1.286, *P* = 0.202), but differed significantly in F2 and F3 (F2: *T*_99.049_ = 3.394, *P* = 0.001; F3: *T*_77.320_ = 3.842, *P* < 0.001). The realized heritabilities of female response to HO estimated both from the high- and low-selected lines were not significantly larger than zero (Table 1, Additional file 2: Table S2).

### Discussion

In this study, the area of male orange coloration became significantly different between high- and low-selected lines after the three generations of artificial selection. This indicates that the differences in the frequencies of alleles at loci affecting the orange area between the lines increased over the generations through selection. The realized heritability estimated from male orange area in high- and low-selected lines was 1.47 and 0.32, respectively, consistent with values in a previous study (0.20–1.50) [19]. Since the response to artificial selection and the heritability estimates would depend largely on initial genetic variation, the similar estimates of heritability between the present and previous studies indicates that similar levels of genetic variation for male orange area might be maintained in the populations in Okinawa and Trinidad. The heritability estimated from the low-selected line was not significantly larger than zero, indicating that the frequencies of alleles affecting smaller orange area might be larger in the initial populations of the artificial selection.

The female responses to HO also became significantly different between high- and low-selected lines after three generations of artificial selection. This indicates that differences in female response to orange coloration were partly due to allelic differences between selected lines. However, although females that responded to HO males were selected in the high-selected lines, the female response to HO males did not increase over generations (Fig. 1), and the estimated heritability from the high-selected line was not significantly larger than zero. On the other hand, in low-selected lines, the female response decreased over generations and the heritability was marginally significant (0.595, *P* = 0.057). This might be because the initial population included allelic variations for decreasing response, but did not include allelic variations for increasing female response.

Taking the female response to orange coloration in high and low-selected lines as a whole, the heritabilities of female preference estimated in this study were lower than those estimated in a previous studies in the guppy (0.3) [22], and in a study in the three-spined stickleback (*Gasterosteus aculeatus*) (0.43, se = 0.37) [29]. Beside the initial genetic variation for female preference, female responses estimated from exposure to digital images of a male might be different from female preference when presented with real males. Orange spots on real male guppies are more chromatic, and female guppies prefer males based on the combination of chroma, luminance and color pattern [23]. It has been showed that variations in female response to HO males is positively correlated with the expression of multiple opsin genes which are affected by both the light environment and by an allelic difference in the long-wavelength-sensitive 1 (*LWS*-*1*) gene [23]. Thus, it is possible that environmental variation of opsin expression might have caused the lower genetic variation seen in this study.

The present experiments support previous studies showing that variations in male orange area and female preference are partly due to genetic variation, and are reflected in this study’s success in obtaining genetically different lines of males with large and small orange areas. Males in the high- and low-selected lines could be used for QTL-seq for determining candidate genomic regions affecting male orange area.

## Limitations

Feral guppy populations from Okinawa, Japan were used in this study, but the artificial selection experiment involving male orange area has already been tested using Trinidadian guppies. In addition, we did not set up replicates or controls for the artificial selection experiment.

## Supplementary information

**Additional file 1.** Experimental procedure. Detailed description for the measuring of male orange area and female preference, and the artificial selection.

**Additional file 2: Tables S1, S2.** Data for each generation. Fundamental statistics and realized heritabilities in each generation.

**Additional file 3.** Data list of male orange area and female preference. Data of all individuals for which male orange area and female preference was measured.

## Data Availability

All data generated or analyzed during this study are included in this published article and in Additional file 3.

## Abbreviations

HO: High orange: the large and colorful orange spots coloration
LO: Low orange: the small and drab orange spot coloration
LWS: Long-wavelength-sensitive
QTL: Quantitative trait locus
SNP: Single-nucleotide polymorphism
