# Supplementary material for: Genetic segregation for male body coloration and female mate preference in the guppy
Source: BMC Res Notes. 2020 Jan 30;13:49. doi: 10.1186/s13104-020-4909-5 (PMC6993340; doi:10.1186/s13104-020-4909-5)
Supplement: Supplementary file 1 — Additional file 1. Experimental procedure. Detailed description for the measuring of male orange area and female preference, and the artificial selection. [file 13104_2020_4909_MOESM1_ESM.doc]

**Detailed description for measurement of male orange area**

The male orange area was quantified using digital camera images. The males were anesthetized with 2-phenoxyethanol solution, and both the right and left sides of the males photographed using a digital camera (D3300, Nikon, Japan). Distance between the lens of the camera and the male was fixed at 20 cm, and two krypton lamps (25 W) were positioned at fixed distance and angles from the male. The recorded images were analyzed using Photoshop CS4 (Adobe, San Jose). We measured the area of all the orange spots on the body and caudal fin as well as the total area of the body and caudal fin. To standardize orange area by body size, the total area of the orange spots was divided by the total area of the body and caudal fin. The male orange area was calculated as the average of the right and left sides.

**Detailed description for female preference**

Female preference for male orange coloration was estimated as the response to digitally modified video images of a male. Using digital images made it possible to measure female response to the same stimulus over all generations. The two images presented to the females were the same as those used in a previous study [1]. The images were modified from a video of a courting male with large/colorful orange spots (relative orange spot area: 14%, saturation: 91%, high-orange, HO), and of another with small/drab orange spots (relative orange spot area: 9%, saturation: 41%, low-orange, LO). The total lengths of the males in both images were adjusted to 24 mm on the display monitor.

A focal female was introduced into the test aquarium (18 D × 30 W × 17 H cm) with 1 cm gravel on the bottom and 9 cm water depth. A color liquid crystal display monitor (FlexScan L367; Eizo) was placed 2 cm from the long side of the aquarium to display the two digitally modified videos. A metal halide lamp (MF400DL/BUDP; Iwasaki Electric Co.) illuminated the test aquarium from 60 cm above the water surface. The focal females were given 5 min to acclimatize to the test aquarium during which time they were shown two blank aquarium images (11 W × 7 H cm) on the right and left sides of the monitor. Then, the digital images of the HO male and LO male were presented to the female for 5 min, and the female’s behavior monitored using a digital video camera (HDR-CX630V; Sony). We measured the time that the focal female was facing the images and swimming within 5 cm of each male image. To eliminate any bias effects, a second trial was performed 24 h after the first trial in which the same male images were presented to the same test female on the opposite sides from the first trial. Finally, from the two measurements, we calculated the sum of time that females spent on the side of the aquarium displaying the HO and LO male images, and regarded the proportion of time that the females spent on HO male image to total time that females spent on the HO and LO male images as female response to large/colorful orange spots.

**Selection of individuals for Artificial selection**

In choosing individuals for artificial selection, females that included any of the following three patterns were excluded: (1) proportion of response to colorful male image was 1 or 0; (2) no response to either male image in either the first or the second trial; (3) the total time of response to both male images was <15 s. In addition, for both males and females, individuals were selected not to contain more than two siblings from the same brood and not to contain individuals of both selected lines from the same brood.

**Reference**

1. Sakai Y, Kawamura S, Kawata M. Genetic and plastic variation in opsin gene expression, light sensitivity, and female response to visual signals in the guppy. Proc Nat Acad Sci. 2018; 115: 12247-12252.
