# Supplementary material for: Genetic segregation for male body coloration and female mate preference in the guppy
Source: BMC Res Notes. 2020 Jan 30;13:49. doi: 10.1186/s13104-020-4909-5 (PMC6993340; doi:10.1186/s13104-020-4909-5)
Supplement: Supplementary file 2 — Additional file 2: Tables S1, S2. Data for each generation. Fundamental statistics and realized heritabilities in each generation. [file 13104_2020_4909_MOESM2_ESM.doc]

**Additional table S1. Mean ± SD of male orange area and female preference in the selection lines each generation.**

|  | Generation | Line | N | Mean | SD |
| --- | --- | --- | --- | --- | --- |
| Male orange area | P | Base | 85 | 6.804 | 3.155 |
|  | F1 | High | 53 | 9.958 | 6.248 |
|  |  | Low | 47 | 5.365 | 2.083 |
|  | F2 | High | 54 | 10.120 | 3.360 |
|  |  | Low | 51 | 4.893 | 1.832 |
|  | F3 | High | 45 | 14.344 | 5.165 |
|  |  | Low | 45 | 5.743 | 2.538 |
| Female preference | P | Base | 76 | 0.620 | 0.229 |
|  | F1 | High | 47 | 0.554 | 0.175 |
|  |  | Low | 45 | 0.500 | 0.223 |
|  | F2 | High | 51 | 0.559 | 0.141 |
|  |  | Low | 52 | 0.470 | 0.125 |
|  | F3 | High | 39 | 0.590 | 0.152 |
|  |  | Low | 45 | 0.468 | 0.137 |
| Sample size of female preference are less than number of females that measured, because individual that total time of response to both male image is 0 can not calculate proportion of response to HO image. | | | | | |

**Additional table S2.** Realized heritability (± SEM) of male orange area and female preference in the selected lines each generation

|  | *h^2^* _High_ | | | | | |  | *h^2^* _Low_ | | | | | |
| --- | --- | --- | --- | --- | --- | --- | --- | --- | --- | --- | --- | --- | --- |
| Male orange area | |  |  |  |  |  |  |  |  |  |  |  |  |
| P-F1 | **1.392** | ± | 0.225 | **1.472** | ± | 0.243 |  | **1.012** | ± | 0.195 | 0.315 | ± |  |
| F1-F2 | 0.100 | ± | 0.591 |  |  |  |  | 0.484 | ± | 0.212 |  |  | 0.111 |
| F2-F3 | **3.440** | ± | 0.682 |  |  |  |  | **-0.873** | ± | 0.199 |  |  |  |
| Female preference | |  |  |  |  |  |  |  |  |  |  |  |  |
| P-F1 | -0.615 | ± | 0.171 | -0.122 | ± | 0.120 |  | **1.116** | ± | 0.259 | 0.595 | ± |  |
| F1-F2 | 0.066 | ± | 0.231 |  |  |  |  | 0.306 | ± | 0.248 |  |  | 0.148 |
| F2-F3 | 0.563 | ± | 0.261 |  |  |  |  | 0.047 | ± | 0.233 |  |  |  |
| Value in bold indicate significance (*P* < 0.05). | | | | | | | | | | | | | |
